# Voxel-Based Sensitivity of Flat-Panel CT for the Detection of Intracranial Hemorrhage: Comparison to Multi-Detector CT

**DOI:** 10.1371/journal.pone.0165794

**Published:** 2016-11-02

**Authors:** Andreas M. Frölich, Jan-Hendrik Buhk, Jens Fiehler, Andre Kemmling

**Affiliations:** 1 Department of Diagnostic and Interventional Neuroradiology, University Medical Center Hamburg-Eppendorf, Martinistraße 52, 20246, Hamburg, Germany; 2 Department of Neuroradiology, Campus Lübeck, University Medical Center Schleswig-Holstein, Ratzeburger Allee 160, 23538, Lübeck, Germany; "INSERM", FRANCE

## Abstract

**Objectives:**

Flat-panel CT (FPCT) allows cross-sectional parenchymal, vascular and perfusion imaging within the angiography suite, which could greatly facilitate acute stroke management. We hypothesized that FPCT offers equal diagnostic accuracy compared to multi-detector CT (MDCT) as a primary tool to exclude intracranial hemorrhage.

**Methods:**

22 patients with intracranial hematomas who had both MDCT and FPCT performed within 24 hours were retrospectively identified. Patients with visible change in hematoma size or configuration were excluded. Two raters independently segmented hemorrhagic lesions. Data sets and corresponding binary lesion maps were co-registered to compare hematoma volume. Diagnostic accuracy of FPCT to detect hemorrhage was calculated from voxel-wise analysis of lesion overlap compared to reference MDCT.

**Results:**

Mean hematoma size was similar between MDCT (16.2±8.9 ml) and FPCT (16.1±8.6 ml), with near perfect correlation of hematoma sizes between modalities (ρ = 0.95, p<0.001). Sensitivity and specificity of FPCT to detect hemorrhagic voxels was 61.6% and 99.8% for intraventricular hematomas and 67.7% and 99.5% for all other intracranial hematomas.

**Conclusions:**

In this small sample containing predominantly cases with subarachnoid hemorrhage, FPCT based assessment of hemorrhagic volume in brain yields acceptable accuracy compared to reference MDCT, albeit with a limited sensitivity on a voxel level. Further assessment and improvement of FPCT is necessary before it can be applied as a primary imaging modality to exclude intracranial hemorrhage in acute stroke patients.

## Introduction

Current generation C-arm angiography systems for neurointerventional procedures are increasingly equipped with flat-panel detectors capable of performing computed tomography (FPCT) directly within the angiography suite. This emerging imaging modality has proven particularly useful in peri-interventional imaging [1–3], e.g. for position assessment of endovascular implants or detection of hemorrhagic complications. However, its diagnostic accuracy compared to conventional multi-detector CT (MDCT) remains incompletely understood, which currently limits the method’s applicability as a primary diagnostic tool.

In patients with acute stroke, the pivotal role of diagnostic imaging is the exclusion of intracranial hemorrhage[4], which is nowadays routinely performed with cross-sectional imaging. If intracranial hemorrhage could be excluded directly with sufficient certainty within the angiography suite using FPCT instead of a separate imaging session with a CT or MR scanner, endovascular access could be established much faster in patients with ischemic stroke due to intracranial large vessel occlusion who may benefit from mechanical thrombectomy. This would contribute to the overall goal of decreasing onset-to-reperfusion time, which has been established as an important determinant of patient outcome [5, 6]. In conjunction with recent developments in non-invasive vascular imaging and parenchymal blood volume estimation using C-arm systems [7, 8], an adequate acute stroke imaging protocol performed entirely with a C-arm system seems conceivable. However, before this approach can be considered for clinical implementation, the diagnostic accuracy of FPCT needs to be assessed. Previous reports have demonstrated the principal ability of FPCT to depict intracranial hematomas in humans [1, 2, 9] as well as in an animal model [10] while raising concern for a limited sensitivity, particularly related to beam hardening artifacts. However, sufficiently high sensitivity is required to exclude hemorrhage with certainty. To evaluate the diagnostic accuracy of FPCT to detect intracranial hemorrhage in comparison to reference MDCT, we performed a systematic voxel-based classification analysis in co-registered image data sets, hypothesizing that FPCT would be of equal diagnostic accuracy compared to MDCT.

## Materials and Methods

### Study Design

This was a retrospective, multi-center study approved by the Institutional Review Board (Ethikkomission der Ärztekammer Hamburg), Nr. WF-007/14. Individual informed consent was waived by the review board. We consecutively identified patients with imaging examinations acquired at our two university hospitals between July 2011 and July 2014 according to the following inclusion criteria: (1) non-enhanced MDCT of the head and non-enhanced FPCT of the head performed within 24 hours of each other, (2) presence of intracranial hemorrhage (subarachnoid, sub-/epidural, parenchymal and/or intraventricular). To assess for any changes in hematoma size or anatomical configuration, all identified matching CT and FPCT data sets were compared side-by-side by a resident with 4 years experience in neuroimaging. Patients with any observable change in hematoma size or configuration were excluded. The presence of coils, stents or ventricular drains was noted. All scans were performed without immediately preceding contrast administration. We did not exclude patients based on the presence of residual contrast material that was applied preceding the scan, e.g. during neurointerventional procedures.

### Image Acquisition

#### Multidetector CT

Conventional non-enhanced MDCT examinations of the head were obtained with standard protocol from vertex to foramen magnum. Images were routinely reconstructed with a soft-tissue type kernel in the axial plane with 4mm or 5mm slice thickness and increment: 256-slice MDCT scanner (Brilliance iCT; Philips Medical Systems, Best, The Netherlands) with 120 kV, 300 mAs, collimation 0.625mm. 128-slice MDCT scanner (Siemens Somatom AS+, Siemens Healthcare, Forcheim, Germany) with 120 kV, 320 mAs, collimation 0.6mm.

#### Flat Panel CT

In both institutions, FPCT raw data were acquired using an Allura Xper FD 20/20™ angiography system (Philips Healthcare, Best, The Netherlands) with the following acquisition parameters: 20s rotational acquisition, 220° rotation, 617 single frames at a frame rate of 30/s, 48cm detector field of view, 1024 acquisition matrix. Images were reconstructed using a soft-tissue kernel with an isotropic voxel size of 0.9x0.9x0.9mm on a dedicated workstation for FPCT data (XperCT Dual 3.2.0). For image analysis, these isotropic FPCT data were viewed in the axial plane with a slice thickness and increment matching that of the corresponding MDCT examination (4mm or 5mm).

### Image Analysis

Two neuroradiologists with 4 and 11 years of experience independently rated intracranial hematomas and classified hemorrhagic voxels by segmenting hyperdense lesions slice-by-slice in MDCT and FPCT (Analyze 11.0, AnalyzeDirect, Inc., Overland Park, KS, USA), using manual tracing with semiautomated edge detection (range of semiautomated detection was 20 to 120 Hounsfield Units (HU)). Window width and center were adjustable by the raters for optimal visual contrast. Separate region of interest (ROI) categories were defined for intraventricular hematomas and all other intracranial hematomas, hypothesizing that these compartments may be affected to different degrees by artifacts. To minimize recall bias, raters were first presented with MDCT images from the first half of the study population and FPCT images from the second half of the population in randomized order. After a break of 2 weeks, raters were presented with the remaining MDCT and FPCT images in randomized order. MDCT and FPCT images were co-registered using semi-automatic, rigid affine linear transformation with 9 degrees of freedom including shear correction for gantry tilt; Analyze 11.0, AnalyzeDirect, Inc., Overland Park, KS, USA). All ROIs were checked to minimize bias by image quality so that ROI-slices with artifacts related to metal implants were excluded from analysis in consensus. In addition, the extent of intraventricular hemorrhage was assessed by one rater according to the semiquantitative Graeb scale ranging from 0 (no intraventricular hemorrhage) to 12 (completely blood-filled and expanded ventricles) [11].

Total volumes of hematoma in FPCT and MDCT were recorded and compared by the root-mean-square deviation (RMSD). Diagnostic accuracy to detect hematoma in FPCT was calculated with MDCT as the standard of reference. Binary segmented ROIs of hemorrhagic lesions in co-registered reference MDCT and FPCT were compared by voxel wise overlap. The degree of lesion similarity between FPCT and MDCT was calculated using the DICE coefficient (the relative overlap between coregistered segmented hemorrhagic lesions denoted as Lesion_CT_ and Lesion_FPCT_ below).

$$\text{DICE}\;\text{coefficient}=2\times \frac{Lesion_{CT}\cap Lesion_{FPCT}}{Lesion_{CT}+Lesion_{FPCT}}$$

The total number of voxels attributed to true positive (TP), true negative (TN), false positive (FP) and false negative (FN) lesion overlap was counted to calculate sensitivity and specificity for detection of hemorrage in FPCT compared to MDCT. Voxel analysis and co-registration was performed in FSL (FMRIB Software Library v5.0, Oxford, Uk).

The contrast resolution between hemorrhagic voxel values and normal brain parenchyma was determined by the contrast-to-noise ratio (CNR). Because the FPCT data used for our study do not directly report data according to the Hounsfield scale, we calculated the rescale slope and intercept for FPCT images placing a ROI in air and cerebrospinal fluid, and comparing this to identical ROIs in the MDCT to obtain HU in FPCT. For hematoma, the mean HU and standard deviation was determined for voxels in segmented hemorrhagic lesions. For normal brain, the mean and standard deviation of HU was determined in at least four representative regions of interests (ROIs) placed in homogeneously appearing brain parenchyma avoiding volume averaging from blood vessels, sulci, and cisterns. The CNR was then calculated as follows [12]

$$\text{CNR}=\frac{Mean_{lesion}-Mean_{normalbrain}}{\sqrt{{\left(SD_{lesion}\right)}^{2}+}{\left(SD_{normalbrain}\right)}^{2}}$$

### Statistical Analysis

Baseline variables are described using standard descriptive statistics. Voxel-wise inter-rater reliability (agreement of hemorrhagic voxel classification) was assessed by weighted Cohen’s kappa. For univariate comparisons, the independent samples t-test and the Mann-Whitney test as well as the Wilcoxon test for dependent samples were used as appropriate. All statistical analyses were performed using Medcalc 12 (MedCalc Software, Mariakerke, Belgium).

## Results

### Demographic and Clinical Data

Among a total of 38 patients matching the initial criteria, 15 were excluded due to an observed change in hematoma configuration. One patient was excluded because coregistration of MDCT and FPCT data failed, most likely due to severe image artifacts. 22 patients entered the final analysis. Mean age ±SD was 58.4 ± 12.5 years, 14 patients (64%) were females. Of these, 16 patients had subarachnoid hemorrhage while 3 patients underwent acute ischemic stroke intervention with hemorrhagic transformation and 3 patients underwent evaluation of a primary intracerebral hematoma. The mean time difference ±SD between MDCT and FPCT imaging was 472±408 minutes. The median Graeb score for the extent of intraventricular hemorrhage on MDCT was 5.0 (interquartile range 2.5–8.5) and was not significantly different on FPCT (median 4.5; interquartile range 2.0–9.0; p = 0.56).

### Voxel-based analysis

**Fig 1:**
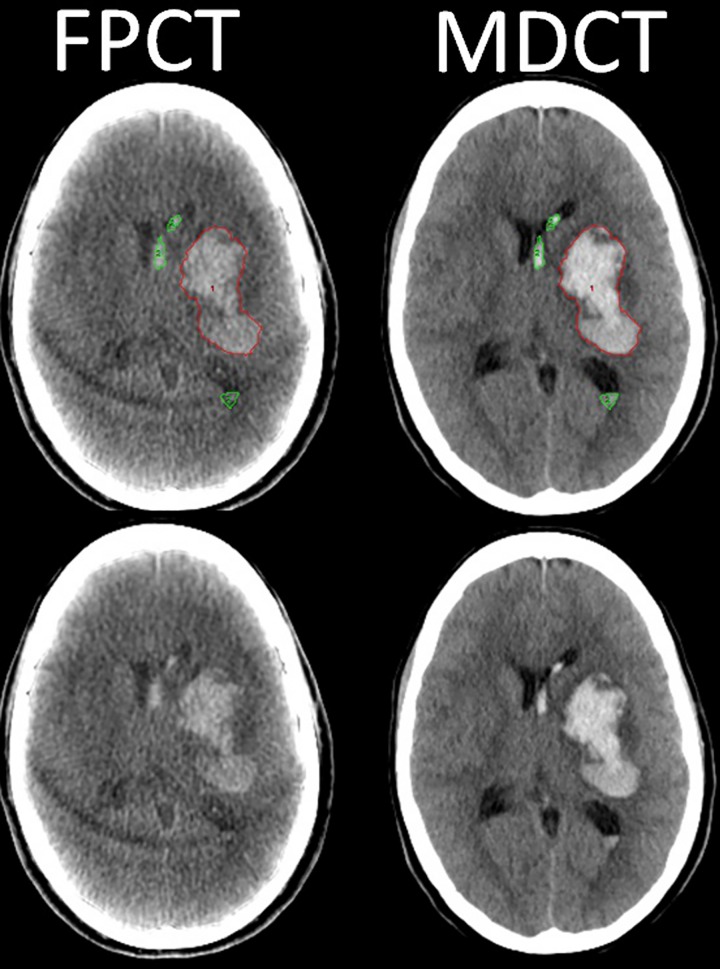
Depiction of intracranial hemorrhage on FPCT and MDCT. Figure shows axial reconstructions from FPCT and MDCT in a patient with intracerebral and intraventricular hemorrhage. In the upper row, regions of interest were drawn on the MDCT image and superimposed onto the co-registered FPCT image. The same images without superimposed regions of interest are shown in the bottom row.

**Fig 2:**
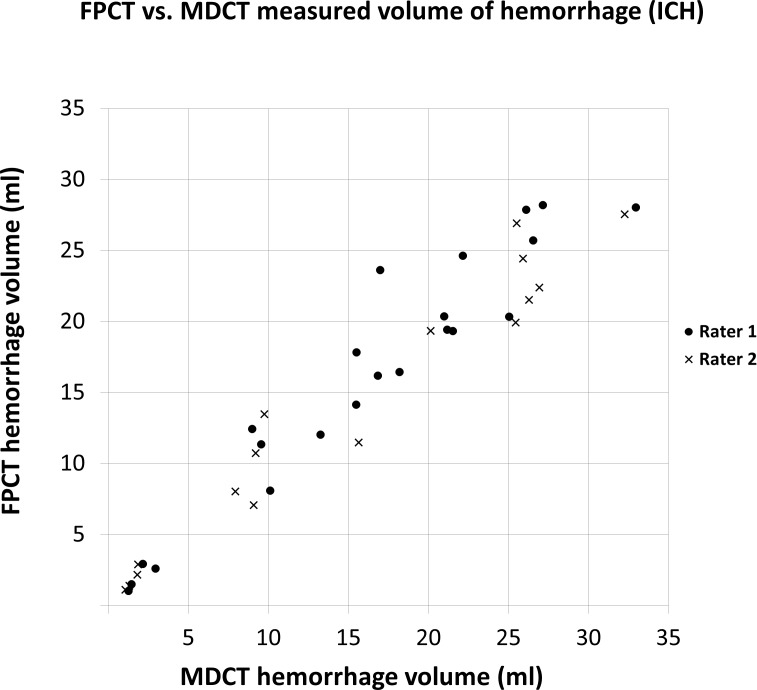
Intracranial hematoma volumes. Diagram shows corresponding hemorrhage volumes measured on FPCT and MDCT for both raters in all patients.

In 22 patients, a total of 36 million intracranial voxels were analyzed in both modalities, respectively. An example of corresponding MDCT and FPCT slices is shown in Fig 1. On MDCT, mean volume ±SD of all intracranial hematomas was 16.2±8.9 ml (range 1.2 to 33.0), on FPCT 16.1±8.6 ml (range 2.7 to 28.2). All hematomas in MDCT were visually detected in FPCT, thus on the patient level, there were no false negative FPCT ratings for presence of hemorrhage in our study cohort. There was near perfect correlation of hematoma size between the two modalities (ρ = 0.95, p<0.001). The RMSD of hematoma volume between FPCT and MDCT was 3.58 ml for all measurements across both raters. Fig 2 demonstrates the assessment of total hematoma volumes for MDCT and FPCT. The mean attenuation value across all hematomas was 73.6±4.1 HU on MDCT and 81.4±8.79 HU on FPCT.

Voxel-based assessment to detect hemorrhage in FPCT demonstrated an average sensitivity, specificity, positive and negative predictive value of 61.3%, 99.7%, 66.2% and 99.6% for intraventricular hematomas and 67.7% and 99.5%, 68.9% and 99.5% for all other intracranial hematomas. Voxel-wise interrater reliability as assessed by ROI segmentation data of the second rater (16 of 22 cases) was substantial (weighted kappa κ = 0.853, 95%CI 0.852–0.854). The RMSD of hematoma volume between raters 1 and 2 was 3.45ml for MDCT and 4.43ml for FPCT. The contrast resolution between hemorrhagic voxels and normal brain parenchyma was 51% lower in FPCT compared to CT (mean CNR 2.4 +/- 0.9 vs. 4.9 +/- 2.0, respectively, P < 0.001) (Table 1).

**Table 1:** Patient characteristics and imaging of hemorrhagic lesions in FPCT compared to MDCT.

Case	Sex	Age	Sensitivity	Specificity	DICE	CNR MDCT	CNR FPCT
1	F	51	62.6%	99.5%	0.63	4.7	1.6
2	F	48	55.9%	99.9%	0.56	1.5	1.6
3	M	29	78.2%	99.4%	0.78	9.4	3.5
4	F	62	56.2%	99.0%	0.56	6.7	2.8
5	F	53	55.3%	99.1%	0.55	5.9	4.5
6	F	75	39.5%	99.9%	0.39	7.1	3.2
7	F	46	48.5%	99.1%	0.49	5.0	1.9
8	F	36	43.0%	99.4%	0.43	4.0	3.3
9	F	53	76.7%	99.4%	0.77	6.3	3.0
10	M	43	65.8%	99.0%	0.66	7.2	2.6
11	F	69	74.5%	99.6%	0.74	6.9	2.3
12	M	66	50.1%	99.9%	0.50	3.1	2.1
13	M	66	51.6%	99.5%	0.52	1.3	2.4
14	F	65	26.4%	98.6%	0.26	3.5	1.4
15	M	76	49.5%	99.6%	0.49	2.1	0.6
16	M	65	60.3%	99.9%	0.60	3.6	1.5
17	M	69	88.9%	99.6%	0.89	4.9	1.9
18	F	73	81.3%	99.6%	0.81	3.8	3.5
19	F	67	87.0%	99.9%	0.87	4.3	2.2
20	M	66	82.9%	99.9%	0.83	6.4	2.2
21	F	48	72.5%	99.8%	0.73	3.9	2.6
22	F	44	93.2%	99.8%	0.93	6.5	1.6

## Discussion

The present study demonstrates a good agreement of FPCT and MDCT for measuring the size of intracranial hematomas with substantial interrater agreement, which confirms the principal diagnostic utility of FPCT for the depiction of intracranial hemorrhage. To our knowledge, this is the first study reporting voxel-wise sensitivity and hematoma volumes measured on FPCT compared to reference MDCT. With an only moderate sensitivity of FPCT, our data confirm the need for further assessment and improvement of the technique before consideration as a primary diagnostic tool in the evaluation of patients with suspected intracerebral hemorrhage.

A particularly interesting prospect is to assess whether FPCT could be capable of safely excluding hemorrhage in acute stroke patients. With the recent strong evidence supporting endovascular therapy in large-vessel occlusion stroke [13–17], an efficient imaging workflow is required to minimize door-to-catheter times. By avoiding separate MR or CT imaging, FPCT as a primary imaging tool could improve the workflow for patients with acute large vessel occlusion stroke. Evaluating FPCT against MDCT for excluding hemorrhage ideally requires large numbers of hemorrhagic (positive) and normal (negative) CT scans within a close time interval. Because the patient numbers for such study data are limited, we chose a voxel based analysis to examine the detectability of hemorrhage at any voxel in FPCT compared to MDCT. This approach revealed a moderate sensitivity of FPCT for detection of intracranial and specifically intraventricular hematomas compared to MDCT (68% and 62%, respectively). This corresponds to the sensitivity of 58% recently reported by Kau et al. in patients examined with FPCT after acute stroke interventions [9]. These results likely demonstrate a currently limited ability of FPCT to depict the full extent and anatomical distribution of intracranial hematomas. However, given the relatively good agreement between the two modalities in the assessment of overall hematoma size, this limited sensitivity could additionally be explained by imperfections in the coregistration process and rater based segmentation imprecision. It can be expected that in contrast to detecting hemorrhage on a voxel level with intermediate sensitivity, a simple binary assessment for presence or absence of hemorrhage on a patient level may yield a higher sensitivity considering that all hematomas in our study cohort were visibly present in FPCT. This type of analysis would require a larger study cohort, however.

Apart from the exclusion of intracranial hemorrhage, further studies are needed to assess how well FPCT is capable of identifying early ischemic changes in brain parenchyma, hyperdense artery signs due to thrombi or other pathology such as venous infarction or intracranial tumors in order to allow acute stroke management decisions. Since volumetric vascular and perfusion imaging within the angiography suite are also becoming available [7, 8], their ability to detect vascular occlusions, the ischemic core and assess collaterals will need to be characterized as these variables are crucial for acute stroke treatment decisions.

A principal limitation affects all current studies comparing the two modalities: images were acquired at different time points in patients with space-occupying intracranial lesions. Thus, changes in the size and location of hematomas are to be expected and compromise current data. We tried to minimize this problem by rigorous side-by-side comparison of all images and exclusion of patients with any observable changes in hematoma configuration. However, subtle changes could still have affected our results. As a drawback, this approach also reduced the size of our sample, which furthermore predominantly consisted of subarachnoid hemorrhage cases. It should be noted that the relatively high specificity observed in our study is partially attributable to the high number of true negative voxels contained within the total brain space (even though we did exclude all voxels of the extracranial compartments from the analysis). A final limitation of our study is its retrospective design, which limits the generalizability of our results, particularly because we did not include patients without intracranial hemorrhage. Interestingly, the analysis done by Kau et al. did include patients without hematomas and despite this difference in methodology, our observed sensitivity of FPCT was similar [9].

Our analysis emphasizes the utility of a voxel-based approach using rigid co-registration for assessing the diagnostic accuracy of imaging modalities. Compared to non-quantitative (e.g. hemorrhage yes/no) or semiquantitative approaches (e.g. Fisher grading for subarachnoid hemorrhage [18]), the voxel-based method offers a quantifiable, objective description of the diagnostic accuracy of FPCT. As a drawback, this approach is heavily dependent on perfect or near-perfect image alignment. Apart from further clinical investigations, voxel-based analyses could be particularly well-suited for phantom or animal studies. Several different aspects of FPCT for hemorrhage detection remain largely unexplored, including the effects of patient positioning, radiation dose, number of angular projections as well as image reconstruction algorithms. Along with further optimizations in detector technology and image reconstructions, the influence of these variables on the detectability of intracranial hematomas should be further assessed.

Another potential application of FPCT is the management of intracranial hemorrhage. Promising recent advances in minimally invasive neurosurgical approaches for the treatment of intracerebral and intraventricular hematomas suggest that these techniques will play a greater role in the future [19, 20]. Given the comparably easy integration of a C-arm system into the operating room environment compared to a fully equipped MDCT scanner, FPCT may be uniquely suited for planning, monitoring and performing minimally invasive procedures in patients with intracranial hemorrhage.

## Conclusion

FPCT shows good agreement with MDCT for measuring the size of intracranial hematomas, despite a limited sensitivity for hematoma detection. Further optimization and evaluation of the technique is required to assess whether it could be useful as a primary diagnostic tool in acute stroke patients.
